# Acute Generalized Exanthematous Pustulosis Following the Administration of Cephalexin: A Case Report and Review of the Literature

**DOI:** 10.7759/cureus.79205

**Published:** 2025-02-18

**Authors:** Yoshihito Mima, Tsutomu Ohtsuka

**Affiliations:** 1 Dermatology, Tokyo Metropolitan Police Hospital, Tokyo, JPN; 2 Dermatology, International University of Health and Welfare Hospital, Tochigi, JPN

**Keywords:** acute generalized exanthematous pustulosis, cephalexin, euroscar score, il-8, t-cells, viral infection

## Abstract

Acute generalized exanthematous pustulosis (AGEP) is a severe cutaneous reaction characterized by the sudden onset of numerous sterile, non-follicular pustules on an erythematous and edematous background, usually associated with fever. AGEP is commonly triggered by medications, including antibiotics, anticancer agents, and hydroxychloroquine, but infections and vaccinations, such as parvovirus B19 and COVID-19 vaccines, have also been implicated. We report a case of AGEP which developed two days after the administration of cephalexin. The pathological findings of subcorneal pustules and European Severe Cutaneous Adverse Reactions (EuroSCAR) score of 9 confirmed the diagnosis of AGEP. To date, only six cases of cephalexin-induced AGEP have been reported. Among these, five cases involved patients without underlying conditions, while one case occurred in a patient with pustular psoriasis undergoing treatment. Although all six cases were diagnosed based on clinical and histopathological findings, our case is the only one in which the EuroSCAR score has been used for definitive diagnosis. Interestingly, viral infections have been suggested as potential triggers for AGEP. In the present case, the patient had pre-existing cold symptoms before taking cephalexin, raising the possibility that a viral infection contributed to AGEP onset. Viral infections are known to induce CD4+ and CD8+ T-cell activation, which in combination with drug exposure may lead to excessive infiltration of inflammatory T-cells into the skin, resulting in increased production of cytokines such as interleukin (IL)-8 and IL-36. This mechanism could explain why AGEP may develop more readily in the presence of a viral infection. Further accumulation of cases and research is needed to clarify the underlying mechanisms.

## Introduction

Acute generalized exanthematous pustulosis (AGEP) is a condition characterized by the acute onset of diffuse edematous erythema, particularly in intertriginous areas and the face, with numerous non-follicular, sterile pustules developing on the erythematous skin. Patients may experience associated burning sensations or pruritus, and approximately 20% of cases present with mucosal involvement, primarily affecting the oral cavity. Mucosal lesions are typically mild and limited to a single site. The majority of AGEP cases present with fever exceeding 38°C and leukocytosis, often accompanied by mild eosinophilia [1]. AGEP is commonly triggered by medications, including antibiotics, anticancer agents, and hydroxychloroquine, typically occurring within a week following exposure to the causative drug [2]. In addition, infections or virus vaccinations, such as parvovirus B19 and COVID-19 vaccination, have also been considered to trigger the development of AGEP [3,4]. Clinically, the pustules of AGEP resolve spontaneously within 4-10 days, often leaving behind desquamation and postinflammatory hyperpigmentation. The prognosis is generally favorable, but in elderly or immunocompromised patients, high fever or secondary bacterial infections can pose life-threatening risks [1].

Several conditions require differential diagnosis from AGEP, including drug reaction with eosinophilia and systemic symptoms (drug-induced hypersensitivity syndrome), toxic epidermal necrolysis, subcorneal pustular dermatosis, generalized pustular psoriasis, bacterial folliculitis, acne, dermatophytosis, varicella, Kaposi varicelliform eruption, Sweet’s syndrome, impetigo, autoimmune blistering diseases, and Behçet’s disease [1]. Histopathologically, AGEP is characterized by spongiform subcorneal or intraepidermal pustules, prominent dermal papillary edema, and perivascular neutrophilic infiltration, with occasional eosinophil degranulation [5,6]. In some cases, perivascular neutrophilic infiltration and keratinocyte necrosis suggestive of vasculitis may be observed. However, epidermal hyperplasia, a hallmark of psoriasis, is typically absent [5,6].

The diagnosis of AGEP is based on clinical presentation and histopathological findings, with the European Severe Cutaneous Adverse Reactions (EuroSCAR) scoring system serving as a useful diagnostic tool [7]. EuroSCAR score contains the category such as morphology (pustules, erythema, distribution, desquamation postpustulation), course (mucosal involvement, acute onset, resolution, fever, white blood cell count), and histopathology (skin biopsy). A EuroSCAR score of 8 or higher is considered positive and confirms the diagnosis of AGEP [7]. Additional tests such as patch testing, drug-induced lymphocyte stimulation tests (DLSTs), and intradermal testing may be considered as needed [2]. The pathogenesis of AGEP involves multiple immune pathways. Activated CD4+ and CD8+ T-cells respond to specific drug-related triggers, inducing keratinocyte apoptosis and tissue destruction via perforin/granzyme B and Fas ligand pathways, leading to vesicle formation. Additionally, interleukin (IL)-8 or IL-36 secretion promotes neutrophil recruitment, contributing to pustule formation and peripheral neutrophilia [8]. The first-line treatment for AGEP is topical corticosteroid therapy and immediate discontinuation of the causative drug. In severe cases, oral prednisolone is recommended, while supportive therapy with antipyretics and antihistamines can be beneficial. If systemic corticosteroid therapy is ineffective, cyclosporine may be considered as an alternative treatment [2].

Cephalexin is a first-generation cephalosporin beta-lactam antibiotic that exhibits bactericidal activity by inhibiting penicillin-binding proteins [9]. It has strong activity against gram-positive bacteria but limited efficacy against gram-negative bacteria [10]. Adverse effects include dyspepsia, gastritis, diarrhea, abdominal pain, and urticaria, and it is commonly used for the treatment of otitis media and pharyngitis [10,11].

Herein, we report a case of cephalexin-induced AGEP occurring in the context of viral symptoms, while also reviewing previously reported cases.

## Case presentation

**Figure 1:**
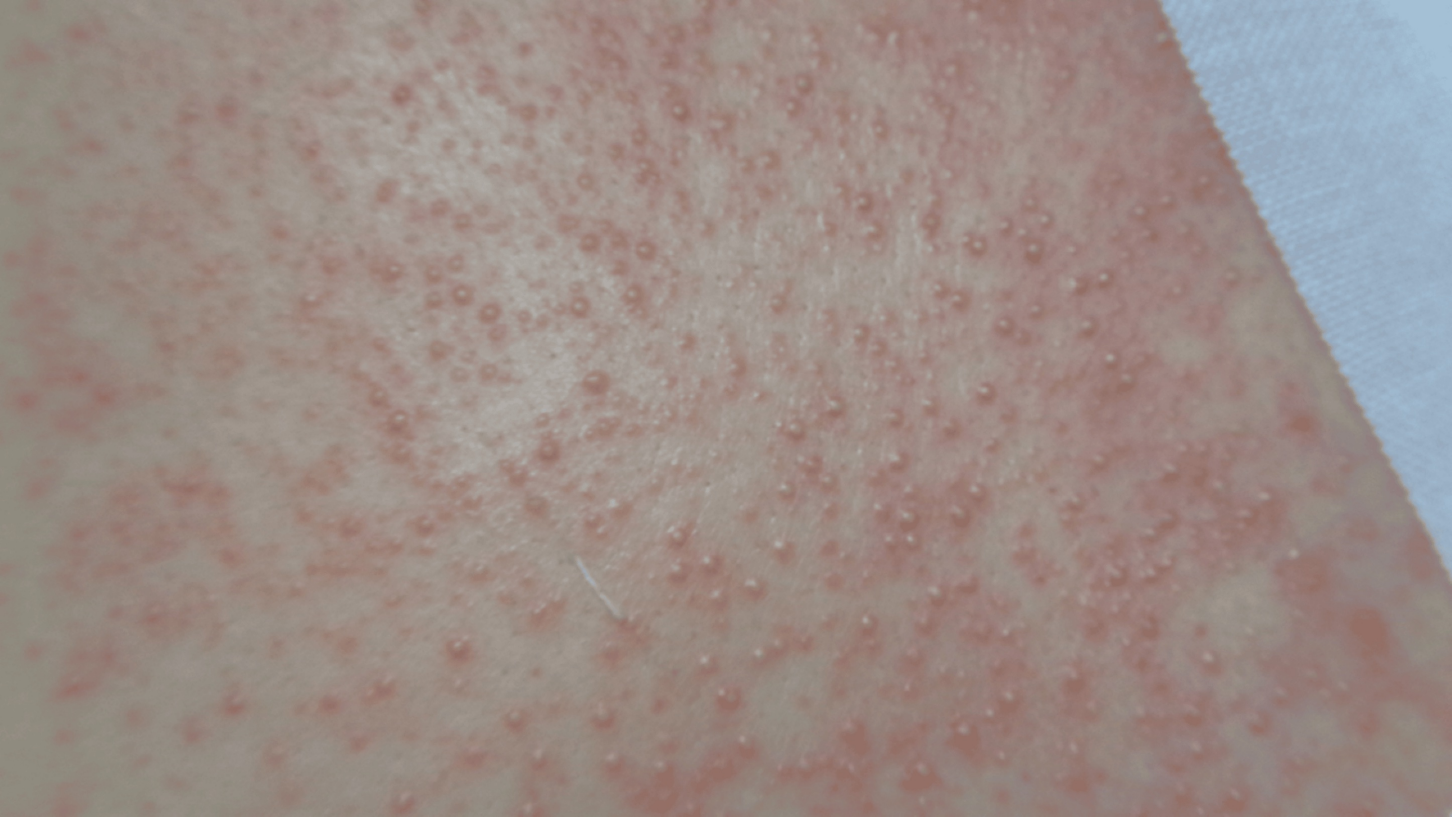
Clinical photograph: Numerous small pustules with erythematous halos were widely distributed across the trunk and extremities, independent of hair follicles.

A 47-year-old male with no significant medical history and no regular medication or supplement use underwent the excision of an epidermal cyst on his back at the Department of Plastic Surgery. Postoperatively, he was prescribed oral cephalexin and topical gentamicin sulfate for the prevention of postoperative infection. Although he had experienced mild upper respiratory symptoms, including cough and nasal discharge, a few days before the procedure, he had no fatigue or fever at that time. On postoperative day 2, while his respiratory symptoms were improving, he developed fatigue, low-grade fever (37.4°C), and a skin eruption, prompting a visit to our Dermatology Department. Physical examination revealed numerous non-follicular pustules with halos on an erythematous base, distributed across the trunk and extremities (Figure 1).

**Figure 2:**
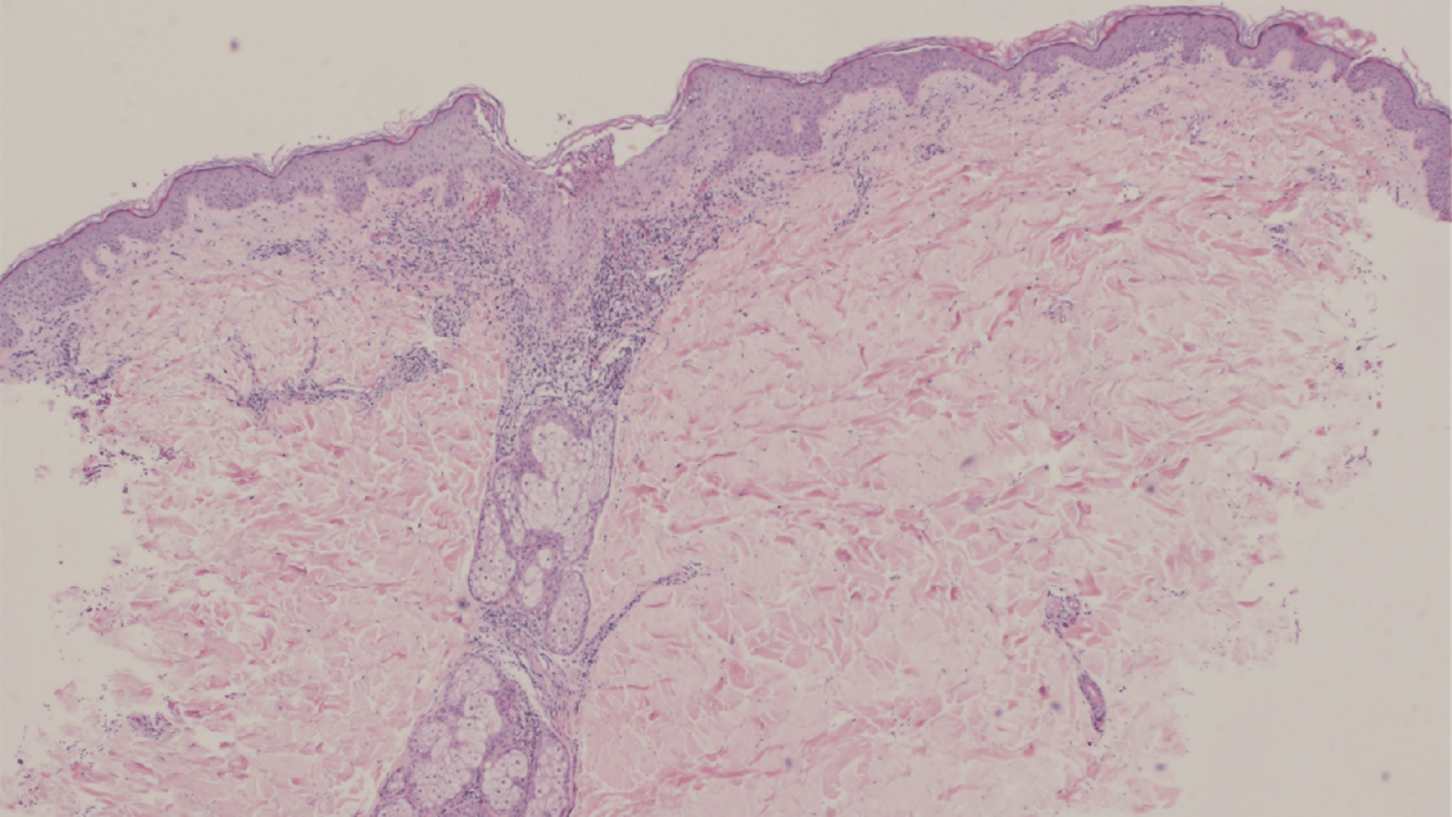
Histopathological examination: Subcorneal pustules were observed, and there was marked infiltration of inflammatory cells in the dermis. Mild edema in the papillary dermis was observed (HE staining; x40). HE: Hematoxylin and eosin

**Figure 3:**
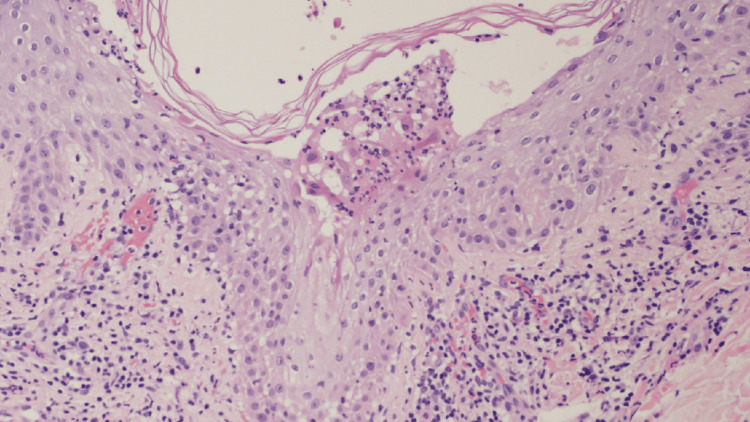
Histopathological examination: A large number of neutrophils were observed within the subcorneal pustules. There was marked lymphocytic infiltration into the epidermis at the basal layer, along with liquefactive degeneration. Additionally, there was prominent infiltration of lymphocytes, neutrophils, and eosinophils around the dermal blood vessels (HE staining; x200). HE: Hematoxylin and eosin

Despite persistent fatigue, his respiratory symptoms had resolved, and no gastrointestinal symptoms were present. Laboratory examinations revealed elevated white blood cells (11,200/μL; reference range, 4,000-10,000/μL) and C-reactive protein levels (1.39 mg/dL; reference range, 0.00-0.30 mg/dL), while all other biochemical and hematological parameters were within normal ranges. Moreover, viral antibody titers were not tested due to the patient's preference. Histopathological examination of the pustular lesions revealed subcorneal pustules filled with numerous neutrophils. Furthermore, there was marked lymphocytic infiltration into the epidermis at the basal layer, along with liquefactive degeneration. Mild edema in the papillary dermis was observed. Additionally, there was a prominent infiltration of lymphocytes, neutrophils, and eosinophils around the dermal blood vessels (Figures 2-3).

Bacterial cultures of the pustules were negative. The patient’s EuroSCAR score was 9 (Table 1), confirming the diagnosis of AGEP.

**Table 1:** EuroSCAR score of the patient EuroSCAR: European Severe Cutaneous Adverse Reactions

Criteria	Description	Score
Morphology		
Pustules	Typical	2
Erythema	Typical	2
Distribution	Typical	2
Postpustular desquamation	No	0
Course		
Mucosal involvement	Yes	0
Acute onset (<24 hours)	Yes	0
Resolution of pustules and erythema (<15 days)	Yes	0
Fever (>38℃)	No	0
Blood neutrophil count (>7000 μ/L)	Yes	1
Histopathology		
Skin biopsy	Subcorneal pustule with mild edema	2
Total score	-	9

Based on the clinical course, cephalexin was identified as the likely causative agent. The patient was treated with topical clobetasol propionate and oral olopatadine, leading to complete resolution of the skin eruption within two weeks. Although patch testing and DLST of the causative drug were recommended to confirm an allergy to cephalexin, the patient declined further testing. No recurrence of skin lesions was observed during a one-month follow-up period, after which both topical and oral treatments were discontinued and follow-up care was completed.

## Discussion

To the best of our knowledge, only six cases of AGEP due to the administration of cephalexin have been documented (Table 2) [12-16].

**Table 2:** Six cases of acute generalized exanthematous pustulosis due to cephalexin AGEP: Acute generalized exanthematous pustulosis

Case	Age	Sex	Past medical history	Diagnosis of AGEP	Symptoms	Onset time	Additional allergy test	Treatment for AGEP
DaCunha et al. [12]	35	Woman	None	Clinical course and histopathological findings	Fever, sweating, and fatigue	Three days after intake	None	Oral corticosteroid
Holscher et al. [13]	58	Male	None	Clinical course	Fever, vomiting, diarrhea, and respiratory failure	Five days after intake	None	Oral corticosteroid and topical corticosteroids
Abbas et al. [14]	54	Female	Pustular psoriasis	Clinical course and histopathological findings	Fever, blisters, and epidermal detachment	Two days after intake	None	Oral cyclosporine and topical corticosteroids
Arroyo et al. [15]	47	Female	Unknown	Clinical course and histopathological findings	Unknown	Unknown	None	Topical corticosteroids
Choon et al. [16]	Unknown	Unknown	Unknown	Unknown	Unknown	One day after intake	Unknown	Unknown
Our case	47	Male	None	Clinical course and histopathological findings	Fever and fatigue	Two days after intake	None	Topical clobetasol propionate and oral olopatadine

Among these, five cases involved patients with no underlying conditions who developed AGEP rapidly after taking cephalexin for a skin infection. The remaining case involved a patient undergoing treatment for pustular psoriasis who developed AGEP after cephalexin was prescribed for a concurrent skin infection. Both pustular psoriasis and AGEP are characterized by sterile pustules, and their pathogenesis is linked to T helper 17 (Th17) cell activation [17] and the involvement of cytokines such as IL-8 and IL-36 [18]. In their report, Abbas et al. suggested that patients with pustular psoriasis may have an increased susceptibility to the development of AGEP. All six cases were diagnosed with AGEP based on clinical course and histopathological findings; however, our case is the only one in which the EuroSCAR score has been used for definitive diagnosis. The latency period between cephalexin administration and AGEP onset ranged from one to five days, which is consistent with the typical incubation period of AGEP development [1,2]. Notably, none of the six cases underwent drug lymphocyte hypersensitivity testing or patch testing. These allergy tests have a risk of false-positive or false-negative results [1,2], and in cases where the causative drug can be clearly identified based on clinical course, such tests may not be deemed necessary. Regarding treatment, three of the six reported cases received systemic corticosteroids or cyclosporine, while two cases were successfully managed with topical corticosteroids or oral histamines. Although antibiotics are among the most frequently implicated drugs in the trigger of AGEP [1,2], our review found only six documented cases specifically associated with cephalexin [12-16]. However, it is essential to consider the possibility of unpublished cases or instances where multiple drugs were administered, making it difficult to pinpoint the exact causative agent.

In our case, pre-existing cold symptoms prior to the administration of cephalexin may have contributed to the occurrence of AGEP. Infections, including viral infections, have been implicated in AGEP pathogenesis [2-4]. Calistru et al. reported a case of AGEP triggered by amoxicillin in a patient with concurrent parvovirus B19 infection and suggested that viral infection may have played an important role in AGEP onset alongside drug-induced immune responses [19]. Viral infections are known to activate CD4+ and CD8+ T-cells in the body [20]. When a causative drug is administered during an ongoing viral infection, increased infiltration of inflammatory CD4+ and CD8+ T-cells into the skin may lead to enhanced production of inflammatory cytokines such as IL-8 and IL-36, potentially triggering AGEP [2-4,8,20]. In the present case, cephalexin was administered in the presence of viral symptoms, which may have resulted in excessive activation of CD4+ and CD8+ T-cells due to the viral infection, coupled with an immune response to the drug, ultimately leading to AGEP onset. This suggests that in addition to the drug itself, viral infection may have played a contributory role in the development of AGEP in the present case.

Herein, we detailed a case of cephalexin-induced AGEP occurring in the context of viral symptoms and reviewed six previously reported cases. While the EuroSCAR score is a valuable tool for quantitatively confirming AGEP diagnosis, its clinical application remains limited. Greater awareness and broader adoption of the EuroSCAR scoring system are warranted. Furthermore, while AGEP can be triggered by viral infections or interactions between viral infections and causative drugs, its precise mechanisms remain unclear. Therefore, further studies and case accumulations are needed to elucidate these pathophysiological pathways.

## Conclusions

AGEP is a severe drug eruption characterized by the sudden onset of numerous small pustules accompanied by edematous erythema. The mainstay of treatment is discontinuation of the causative drug and topical corticosteroids, with rapid resolution typically observed within two weeks. Herein, we presented a case of cephalexin-induced AGEP and reviewed six previously reported cases associated with cephalexin. Since antibiotics are among the most common causative agents of AGEP, careful monitoring of antibiotic administration history is crucial when AGEP is suspected. A limitation of our study is that viral antibody testing was not performed. However, as AGEP developed following symptoms suggestive of a viral infection, a possible association between viral infection and AGEP onset cannot be ruled out. AGEP may be triggered by viral infection alone or by an interaction between viral infection and the causative drug. Therefore, further case accumulation and research are essential to elucidate its underlying mechanisms.

## References

[1] Tetart F, Walsh S, Milpied B (2024). Acute generalized exanthematous pustulosis: European expert consensus for diagnosis and management. J Eur Acad Dermatol Venereol.

[2] Parisi R, Shah H, Navarini AA, Muehleisen B, Ziv M, Shear NH, Dodiuk-Gad RP (2023). Acute generalized exanthematous pustulosis: clinical features, differential diagnosis, and management. Am J Clin Dermatol.

[3] Lee D, Kang JN, Hwang SH, Lee YS, Kim H, Seo JK, Sung HS (2014). Acute generalized exanthematous pustulosis induced by parvovirus B19 infection. Ann Dermatol.

[4] Kang SY, Park SY, Kim JH, Lee SM, Lee SP (2021). COVID-19 vaccine-induced acute generalized exanthematous pustulosis. Korean J Intern Med.

[5] Burrows NP, Russell Jones RR (1993). Pustular drug eruptions: a histopathological spectrum. Histopathology.

[6] Beylot C, Doutre MS, Beylot-Barry M (1996). Acute generalized exanthematous pustulosis. Semin Cutan Med Surg.

[7] Sidoroff A, Halevy S, Bavinck JN, Vaillant L, Roujeau JC (2001). Acute generalized exanthematous pustulosis (AGEP) - a clinical reaction pattern. J Cutan Pathol.

[8] Kabashima R, Sugita K, Sawada Y, Hino R, Nakamura M, Tokura Y (2011). Increased circulating Th17 frequencies and serum IL-22 levels in patients with acute generalized exanthematous pustulosis. J Eur Acad Dermatol Venereol.

[9] Williamson R, Collatz E, Gutmann L (1986). Mechanisms of action of beta-lactam antibiotics and mechanisms of non-enzymatic resistance. (Article in French). Presse Med.

[10] Donowitz GR, Mandell GL (1988). Drug therapy. Beta-lactam antibiotics (2). N Engl J Med.

[11] Thienvibul C, Vachiramon V, Chanprapaph K (2015). Five-year retrospective review of acute generalized exanthematous pustulosis. Dermatol Res Pract.

[12] DaCunha M, Moore S, Kaplan D (2018). Cephalexin-induced acute generalized exanthematous pustulosis. Dermatol Reports.

[13] Holscher CM, Mauck SK, Armstrong L, Buchanan JA (2011). Man with rash and nausea. Acute generalized exanthematous pustulosis after cephalexin use. Ann Emerg Med.

[14] Abbas M, Holfeld K, Desjardins D, Zimmer J (2014). Pustular psoriasis complicated with acute generalized exanthematous pustulosis. J Dermatol Case Rep.

[15] Arroyo MP, Heller P, Pomeranz MK (2002). Generalized pustules in a healthy woman. J Drugs Dermatol.

[16] Choon SE, Der YS, Lai NL, Yu SE, Yap XL, Nalini NM (2018). Clinical characteristics, culprit drugs and outcome of patients with acute generalised exanthematous pustulosis seen in Hospital Sultanah Aminah, Johor Bahru. Med J Malaysia.

[17] Teraki Y, Tanaka S, Hitomi K, Izaki S (2010). A case of generalized psoriasiform and pustular eruption induced by infliximab: evidence for skin-homing Th17 in the pathogenesis. Br J Dermatol.

[18] Marrakchi S, Guigue P, Renshaw BR (2011). Interleukin-36-receptor antagonist deficiency and generalized pustular psoriasis. N Engl J Med.

[19] Calistru AM, Lisboa C, Cunha AP, Bettencourt H, Azevedo F (2012). Acute generalized exanthematous pustulosis to amoxicillin associated with parvovirus B19 reactivation. Cutan Ocul Toxicol.

[20] Rouse BT (1996). Virus-induced immunopathology. Adv Virus Res.

